# Engineered Fungus *Thermothelomyces thermophilus* Producing Plant Storage Proteins

**DOI:** 10.3390/jof8020119

**Published:** 2022-01-26

**Authors:** Larissa Balabanova, Aleksandra Seitkalieva, Yulia Yugay, Tatiana Rusapetova, Lubov Slepchenko, Anna Podvolotskaya, Margarita Yatsunskaya, Elena Vasyutkina, Oksana Son, Liudmila Tekutyeva, Yury Shkryl

**Affiliations:** 1 G.B. Elyakov Pacific Institute of Bioorganic Chemistry, Far Eastern Branch of the Russian Academy of Sciences, Prospect 100-letya Vladivostoka 159, 690022 Vladivostok, Russia; balaban@piboc.dvo.ru (L.B.); sasha0788@inbox.ru (A.S.); Slepchenko.lubov@gmail.com (L.S.); 2 Federal Scientific Centre of the East Asia Terrestrial Biodiversity, Far Eastern Branch of the Russian Academy of Sciences, Prospect 100-letya Vladivostoka 159, 690022 Vladivostok, Russia; yuya1992@mail.ru (Y.Y.); avramenko.dvo@gmail.com (T.R.); subtilita@gmail.com (M.Y.); levina@biosoil.ru (E.V.); 3 Department of Bioeconomy and Food Security, Far Eastern Federal University, B417 Office, Bldg. 20, Ajax St., Russky Island, 690950 Vladivostok, Russia; apodvolot7777@mail.ru (A.P.); son.om@dvfu.ru (O.S.); tekuteva.la@dvfu.ru (L.T.); 4 ARNIKA, Territory of PDA Nadezhdinskaya, Centralnay St. 42, 692481 Volno-Nadezhdinskoye, Primorsky Krai, Russia

**Keywords:** filamentous fungi, *Myceliophthora* *thermophila*, mycoprotein, recombinant protein, maize alpha-zein, amaranth albumin A1

## Abstract

An efficient *Agrobacterium*-mediated genetic transformation based on the plant binary vector pPZP-RCS2 was carried out for the multiple heterologous protein production in filamentous fungus *Thermothelomyces* *thermophilus* F-859 (formerly *Myceliophthora* *thermophila* F-859). The engineered fungus *Th.* *thermophilus* was able to produce plant storage proteins of *Zea* *mays* (α-zein Z19) and *Amaranthus* *hypochondriacus* (albumin A1) to enrich fungal biomass by valuable nutritional proteins and improved amino acid content. The mRNA levels of *z19* and *a1* genes were significantly dependent on their driving promoters: the promoter of tryptophan synthase (P*trpC*) was more efficient to express *a1*, while the promoter of translation elongation factor (P*tef*) provided much higher levels of *z19* transcript abundance. In general, the total recombinant proteins and amino acid contents were higher in the P*tef*-containing clones. This work describes a new strategy to improve mycoprotein nutritive value by overexpression of plant storage proteins.

## 1. Introduction

Most feeds used in agriculture contain either a low level of digestible proteins or poor essential amino acid content [1,2]. This deficit is covered by an increase in the production of fishmeal and dried dairy products or plant protein contained in agricultural fodder crops, such as grain or alfalfa. There are many alternative sources of plant protein for livestock rations. These include oilseeds, byproducts of food production, arable and forage legumes [1,3,4,5]. Many of the available conventional rations are based on agro-industrial byproducts such as cottonseed cake or corn gluten feed and meal, derived from *Zeya mays* grains [3,4,5,6]. The corn gluten has a protein content about 20–60% and a fiber up to 10% [5,6]. However, a small amount of Lys, an absence of Trp and many Leu make the amino acid (AA) composition of maize proteins (zeins) even more unbalanced [7]. Alternatively, *Amaranthus hypochondriacus* seed proteins, most of them being albumins, have the highest essential AA levels and balanced content [5,8,9]. The grain amaranth seeds contain an average of 13–19% crude protein (90% digestibility), with a high Lys and Trp content, 5.7–16.7% fat and 2.5–14% fiber, which are higher than most cereals. Moreover, the pseudocereals are gluten-free, which makes them attractive food and feed alternatives against coeliac disease and gluten sensitivity [9]. Overexpression of an amaranth seed albumin in tuber tissues of regular and sweet potatoes has improved a nutritional value of the transgenic plants [10,11].

Current feed formulation and feed evaluation are generally based on average table values for the contents and digestibility of AAs in the individual feedstuffs [2]. It is frequently necessary to reach the balanced AA composition (near ideal AAs profile) of the microbial protein produced in the digest tract and a feed protein, which has escaped degradation, by supplementing the crystalline source of L-Lys, L-Val, L-Thr, L-Phe, DL-Met, L-Ile, L-His, L-Trp, and L-Leu in a diet [2,12]. However, the cost of balanced rations is very high, and livestock production becomes uneconomical due to the high price of these feed ingredients [1]. Thus, the problem of meeting the world protein demand for food and feed remains. The demand in crop proteins is higher than the total protein production from total worldwide arable land, which can currently produce 600 million tones protein (from leaves and seeds) [5,13].

A protein produced in microbial cells of bacteria, fungi or algae (single-cell protein) can be used to improve the protein content in animal feed [1,5,14,15,16]. Of the various methods available to producers, supplementation of deficient nutrients in the form of biomass after enzymatic treatment of lignocellulolytic fungi, combined with chemical and physical treatments, has been shown to be effective for utilization of low-quality forages, such as cereal straws and other agro-industrial wastes [14]. Fungi contain up to 30–50% protein with an increased Thr and Lys content that is in accordance with the Food and Agriculture Organization (FAO) recommendations [15]. They produce essential nutrients, such as thiamine, riboflavin, biotin, niacin, pantothenic acid, pyridoxine, choline, streptogenin, glutathione, folic acid, and p-amino benzoic acid, as well as fiber (cell wall glucans) that contribute to the diet. In addition, the filamentous fungi have a moderate nucleic acid content (7–10%) in comparison with bacteria and yeasts; what is more, yeasts tend to produce undesired hypermannosylated secreted proteins [15,16]. The fungal biomass of *Aspergillus oryzae*, *Neurospora intermedia*, and *Rizhopus oryzae* grown on vinasse and comparable with the fishmeal AA content and profile were suggested as supplementation alternative for fish feeding [17]. The products of commercially available filamentous fungi *Paecilomyces varioti* and *Fusarium venenatum*, obtained with the use of lignocellulosic sugars, provide a good source of the important group-B vitamins and digestible mycoprotein for animal and human consumption, respectively [15]. Dietary protein may also be derived from those secreted by engineered filamentous fungi [16,18,19,20]. However, the edible food grade filamentous fungus, such as *Neurospora intermedia* or *Fusarium venenatum*, grows in a high-cost cultivation medium [21].

The filamentous fungus *Thermothelomyces thermophilus* (formerly *Myceliophthora thermophila*) can utilize cheap plant biomass and has been shown to be safe for large-scale production processes and is successfully used in metabolic engineering [22,23,24,25]. Moreover, thermophilic fungi are a promising source of novel enzymes for cost-efficient industrial applications, including abundant thermostable enzymes for biomass degradation, and as a cell factory to produce chemicals and biofuels from renewable lignocelluloses [23,24]. In particular, *T. thermophilus* strain C1 has been developed for the production of cellulolytic and hemicellulolytic enzymes substance included in the Generally Recognized as Safe (GRAS) list with a wide range of applications in food and feed industries [26].

In the present study, *Th. thermophilus* strain F-859 (WEPL 264A; BKM F-2109, http://www.vkm.ru/rus/Catalogue.htm accessed on 23 January 2022) was modified due to introduction of valuable plant storage proteins from *Z. mays* and *A. hypochondriacus*. The heterologous gene expression under different fungal promoters, recombinant proteins production, and amino acid content in the engineered clones *Th. thermophilus* rF-859 was analyzed.

## 2. Materials and Methods

### 2.1. Gene Cloning and Vector Construction

Seeds of maize (*Zea mays*) and amaranth (*Amaranthus hypochondriacus*) were purchased from the local seed market and germinated under appropriate conditions. Total RNA was isolated from 2-week-old seedlings by using LiCl method as described previously [27]. The first-strand cDNA synthesis was conducted with MMLV RT kit (Evrogen, Moscow, Russia) according to manufacturer’s instruction. To amplify the full-length sequence *z19* of *Z. mays* 19-kDa α-zein B1 (Genbank Acc. No. AF371269), PCR reactions were carried out with gene-specific primers: forward Z19D 5′-ATA GCC ATG GCA GCC AAA ATA TTT TGC-3′ (*Nco*I restriction site is underlined) and reverse Z19R 5′-GAC TCT AGA CCT A*AT GGT GGT GGT GAT GAT G*AA AGA GGG CAC CAC CAA TGA-3′ (*Xba*I restriction site is underlined, 6xHis tag sequence is in italics). To amplify the full-length sequence *a1* of *A. hypochondriacus* seed albumin (Genbank Acc. No. AF491291), we used the following primers: A1D 5′-ATA GCC ATG GCG GGA TTA CCA GTG-3′ (*Nco*I restriction site is underlined) and A1R 5′-GAC TCT AGA CCT A*AT GGT GGT GGT GAT GAT G*GT TGT TGG ATC CCA ATT CTA-3′ (*Xba*I restriction site is underlined, 6xHis tag sequence is in italics). The *Age*I-*Nco*I fragments of DNA encoding the promoters P*trpC* and P*tef* were obtained as previously described [28].

**Figure 1:**
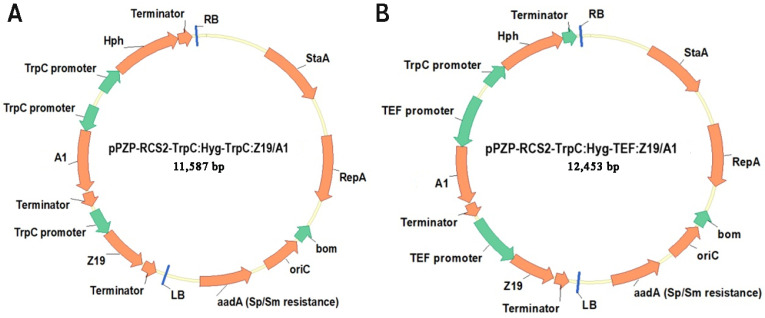
Binary vectors pPZP-RCS2-TrpC:Hyg-TrpC:Z19/A1 (**A**) and pPZP-RCS2-TrpC:Hyg-TEF:Z19/A1 (**B**) carrying genes for synthesis of Z19 and A1 under the control of P*trpC* and P*tef* promoters.

Plasmids pSAT1-MCS and pSAT4-MCS both containing the tandem cauliflower mosaic virus (CaMV) 35S promoter, tobacco etch virus (TEV) leader and the CaMV 35S terminator, were used for construction of expression vectors [29]. The coding sequences *z19* and *a1* were sub-cloned as *Nco*I-*Xba*I fragments into the same sites of the linearized plasmids pSAT1-MCS and pSAT4-MCS, respectively, whereby pSAT1-35S:Z19 and pSAT4-35S:A1 constructions were obtained. Further, the tandem CaMV P*35S* was excised as an *Age*I-*Nco*I fragment and replaced with the *Age*I-*Nco*I fragments of P*trpC* and P*tef* resulting in the constructions pSAT1-TrpC:Z19, pSAT1-TEF:Z19, pSAT4-TrpC:A1, and pSAT4-TEF:A1. The obtained constructs were checked for the absence of mutations by DNA sequencing as described earlier [30]. The pSAT6-TrpC:hph expression cassette carrying hygromycin B phosphotransferase gene *hph* under the control of P*trpC* was obtained previously [28]. The newly constructed TrpC/TEF:Z19, TrpC/TEF:A1, and TrpC:hph expression cassettes were further excised as *Asc*I, I-*Sce*I, and PI-*Psp*I fragments, respectively, and sub-cloned into the same sites of the binary vector pPZP-RCS2 [29]. The final constructions (Figure 1), pPZP-RCS2-TrpC:Hyg-TrpC:Z19/AmA1 and pPZP-RCS2-TrpC:Hyg-TEF:Z19/AmA1, were transferred into *Agrobacterium tumefaciens* strain EHA105/pTiBo542 [31] using GenePulser Xcell^TM^ electroporation system (Bio-Rad Laboratories, Inc., Hercules, CA, USA) in accordance with the manufacturer’s protocol.

### 2.2. Transformation of Th. thermophilus Mediated by Agrobacterium tumefaciens

The *Th. thermophilus* F-859 transformation mediated by *A. tumefaciens* was performed using a modification of the method described previously [22]. A single colony of *A. tumefaciens* harboring binary vector pPZP-RCS2-TrpC:Hyg-TrpC:Z19/AmA1 or pPZP-RCS2-TrpC:Hyg-TEF:Z19/AmA1 was inoculated into LB medium supplemented with 150 μg/mL rifampicin, 200 μg/mL streptomycin, 300 μg/mL spectinomycin and cultivated with shaking at 180 rpm at 28 °C for 12–16 h to an optical density 0.5–1.0 at 600 nm (OD600). The cells were harvested, washed twice with an induction medium (IM) (1× Vogel’s salts, 10 mM glucose, 0.5% glycerol, 40 mM MES, 0.2 mM acetosyringone (AS)), and then diluted to OD600 of 0.15. The culture was grown at 28 °C with shaking at 250 rpm for 6–8 h to OD600 of 0.5–0.8. Fungi were grown on Vogel’s medium in Petri dishes at 45 °C for 15 d for collecting conidia. The plates with conidia were flooded with 10 mL of 0.05% Tween 80 and held during 15 min. Conidia were carefully scraped with bend Pasteur pipette, and then transferred with a 5-mL pipette into a 15-mL tube for centrifugation at 2000 rpm for 2 min to remove mycelium and to collect the precipitated conidia. The supernatant was removed, and conidia were washed by suspending in 5 mL of sterile water, and then adjusted to 1 × 10^6^ conidia per mL with the use of Goryaev camera. An aliquot of 100 μL *A. tumefaciens* culture was mixed with an equal volume of the *Th. thermophilus* conidia 1 × 10^6^ and spread on an IM agar plate (9.0 cm). The spread mixture was covered with an autoclaved nylon blotting membrane (Whatman^®^ Nytran™ N, Maidstone, Kent, UK). After 4 days of co-cultivation at 28, the membrane was transferred onto a potato-dextran agar (PDA) in Petri dishes, supplemented with 50 μg/mL hygromycin B (Thermo Fisher Scientific, Waltham, MA, USA) and 300 μg/mL cefotaxime (Sigma-Aldrich, St-Louis MO, USA), and then cultivated at 45. Putative transformants were visible after approximately 5 days. The colonies were transferred to plates with PDA supplied by hygromycin B (50 μg/mL), and incubated at the same conditions. After selection, the transformants were transferred to the same medium without hygromycin B. Genomic DNA was extracted from randomly selected transformants and wild type mycelia to verify by polymerase chain reaction (PCR) with *hph*-specific primers described previously [28]. Mitotic stability analysis was carried out as described in Supplementary Materials and Methods.

### 2.3. Real-Time PCR Analysis

Total RNA isolation from *Th. thermophilus* cultures and first-strand cDNA synthesis was conducted as described previously [28]. Quantitative PCR (qPCR) analysis was performed using CFX96 thermocycler (Bio-Rad Laboratories) with SYBR green PCR mix (Evrogen, Moscow, Russia). The β-tubulin gene was used as an internal control. The following gene-specific primers were used in qPCR: 5′-GAC CTG CCT GAA ACC GAA CT-3′ and 5′-TCG TCC ATC ACA GTT TGC CA-3′ for *hph* gene; 5′-GGC ACA AAA CAT CAG GGC AC-3′ and 5′-AGA AAT TGC TGG GGG TAG GC-3′ for *z19* gene; 5′-ACA GCA TCA GCC AAT GAA CC-3′ and 5′-TGA TAC GAA GGA CCC ACC AA-3′ for *a1* gene; 5′-CAA GTA TGT CCC TCG TGC C-3′ and 5′-GCC AAA GGG ACC AGC ACG-3′ for β-tubulin gene. Analysis for four biological replicates from four separate RNA extractions and three technical replicates for each qPCR experiment were performed. CFX Manager Software (Version 3.1; Bio-Rad Laboratories, Hercules, CA, USA) was used for data processing. Melting curve analysis was conducted after each run to verify the absence of primer-dimer artefacts or non-specific amplicons.

### 2.4. Protein Isolation and Analysis

Prior to extraction procedures, the 14-day-old mycelia of *Th. thermophilus* were extensively washed with distilled water to remove a residual nutritive medium. The washed tissues were frozen in liquid nitrogen and homogenized. For long-term storage, the samples were kept at −80 °C. A protein concentration in the samples was determined according to the Bradford protein assay [32]. In brief, 15-day-old tissue cultures were frozen and grinded in liquid nitrogen followed by extraction with 5 volumes of buffer containing 20 mM Tris-HCl, pH 8.0, 8 M urea. The homogenates were centrifuged at 14,000× *g* for 20 min and supernatant was used to measure the protein concentration. Bovine serum albumin (New England Biolabs, Ipswich, MA, USA) was used for preparation of the calibration curve. All reagents were purchased from Panreac (Barcelona, Spain) unless specifically denoted.

Extraction of the recombinant storage proteins Z19 and A1 and SDS-PAGE were carried out as described in the paper by Fic et al. [33] (see Supplementary Materials and Methods for details).

### 2.5. Western Blotting

After SDS-PAGE, the proteins were electrotransferred to 0.45-μm nitrocellulose (for Z19 protein) or polyvinylidene fluoride (for A1 protein) membrane (Merck Millipore, Billerica, MA, USA) at 150 V for 2 h using Mini Trans-Blot cell (Bio-Rad Laboratories, Hercules, CA, USA). Total protein normalization was performed using 0.1% Ponceau S in 1% acetic acid solution for 5 min followed by three washes for 5 min in distilled water. Afterwards, the membrane was incubated in I-Block blocking solution (Applied Biosystems™, Foster City, CA, USA) followed by incubation with the mouse anti-6×-His Tag monoclonal antibody (Bialexa, Moscow, Russia) at 1:200 dilution overnight at 4 °C. The membrane was passed through 3 10-min washes in 50 mL of PBS buffer supplemented with 0.02% Tween 20 followed by incubation with the goat anti-mouse IgG secondary antibody at 1:1000 dilution (Invitrogen, Carlsbad, CA, USA) for 1 h at a room temperature. The membrane was developed using 1 mL of 0.25 mM CDP-Star substrate (Thermo Fisher Scientific, Waltham, MA, USA) followed by immediate visualization using Amersham™ ImageQuant™ 800 (Cytiva, Global Life Sciences Solutions, Marlborough, MA, USA).

### 2.6. Determination of Amino Acid Composition

Conidia were placed in liquid potato-dextran medium and cultivated at 45 °C for 14 or 28 days. The fungal biomass was carefully collected by centrifugation at 11,000 rpm for 15 min, transferred into a porcelain bowl, and ground in liquid nitrogen until homogeneous state. Then, the mixture was disintegrated by sonication, dried in a porcelain bowl at 100 °C, and triturated until a powdery state. The sample hydrolysis and quantification of each AA with the use of chromatograph LC-20 Prominence (Shimadzu, Kyoto, Japan) is described in detail in the Supplementary Materials and Methods. The results were calculated and visualized in MS Excel 2010.

### 2.7. Microscopy

Morphological characterization of fungal hyphae was performed on Carl Zeiss AxioSkop 40 equipped with AxioCam HRc color CCD camera. The images were processed and analyzed by AxioVs40 V 4.8.2.0 (Carl Zeiss, Jena, Germany).

### 2.8. Statistical Analysis

All values are expressed as the mean ± standard error. For statistical evaluation, the Student’s *t* test was used to compare the two independent groups. For comparison among multiple data, analysis of variance (ANOVA) followed by a multiple comparison procedure was employed. Fisher’s protected least significant difference (PLSD) *post-hoc* test was employed for the inter-group comparison. The level of statistical significance was set at *p* < 0.05.

## 3. Results and Discussions

### 3.1. Characterization of Transgenic Th. thermophilus rF-859

The binary vector pPZP-RCS2 was used as a backbone to engineer a genetic system allowing simultaneous transferring into the filamentous fungus *Th. thermophilus* F-859 several coding sequences (CDSs), particularly for a selection marker and the valuable plant storage proteins: 19-kDa α-zein B1 from *Z. mays* (Z19) and seed albumin A1 from *A. hypochondriacus* (A1) (Figure 1).

The transcript level of α-zein B1 is highest among the expressed seed proteins in the maize endosperm [34]. Meanwhile, the amaranth seed proteins abundant in albumins are known to possess near ideal AA profiles [8,35].

In each doubly function vector, each gene was placed under the control of constitutive promoters of either the translation elongation factor (P*tef*) from *Aureobasidium pullulans* or tryptophan biosynthesis pathway (P*trpC*) from *Aspergillus nidulans* (Figure 1). These promoters were effectively used in a number of genetic constructs for developing plasmid-based transformation or genome-editing CRISPR/Cas9 and heterologous gene expression systems for fungi, including filamentous thermophile *Th. thermophilus* [22,24,28,36].

**Figure 2:**
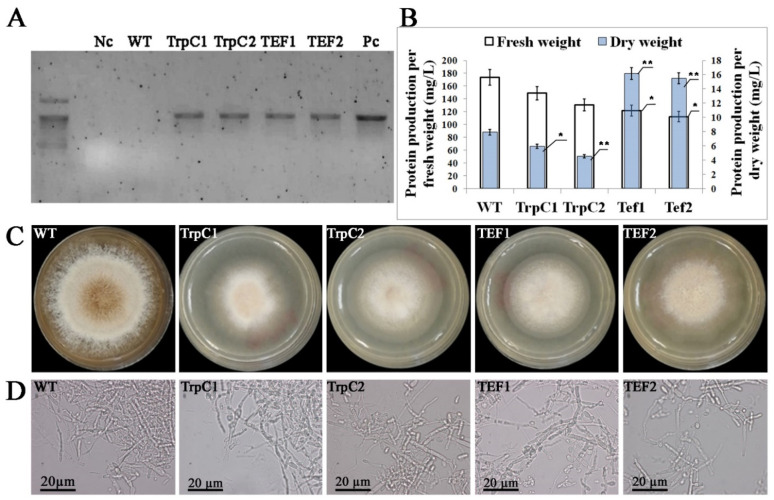
Characterization of transgenic *Th. thermophilus* rF-859 strains. (**A**)—Agarose gel electrophoresis of the *hph* gene amplified using DNA from the *Th. thermophilus* transformants (Nc—negative control (water); WT—wild type strain *Th. thermophilus* F-859; TrpC1 and TrpC2—*Th. thermophilus* rF-859/TrpC:Z19/A1 clones; TEF1 and TEF2—*Th. thermophilus* rF-859/TEF:Z19/A1) clones. (**B**)—Total protein production in *Th. thermophilus* rF-859/TEF:Z19/A1 and *Th. thermophilus* rF-859/TrpC:Z19/A1 mycelia (production was calculated separately for fresh and dried mycelial biomass in 1 L of media). (**C**)—Phenotypic view of the wild type and transgenic strains. (**D**)—Microphotographs of the wild type and *Th. thermophilus* rF-859 hyphae. * *p* < 0.05, ** *p* < 0.01 as compared to wild type strain, Student’s *t*-test.

Two hygromycin (*hph*) resistant recombinant clones of *Th. thermophilus* rF-859 harboring the *z19* and *a1* genes driven by P*trpC* (named TrpC1 and TrpC2), as well as two clones expressing the same genes regulated by P*tef* (named TEF1 and TEF2) were randomly selected for the further study. The successful integration of T-DNA was confirmed by PCR analysis of *hph* in the DNA samples from TrpC and TEF clones (Figure 2A).

All four selected transformants were shown to be mitotically stable after five serial subcultures on a nonselective medium. Thus, our results indicate that this transformation system could be used as an appropriate tool for gene function studies and simultaneous expression of several heterologous proteins in micromycetes. In general, the growth and other phenotypic traits of the transgenic *Th. thermophilus* rF-859, particularly a total protein yield, were not significantly affected by the transformation (Figure 2B, Supplementary Table S1). However, the 3-day old wild type strain was of a larger diameter of the colony (about 3.6 cm (WT), 2.4 cm (TrpC1), 2.7 cm (TrpC2), 2.4 cm (TEF1), and 2.5 cm (TEF2)) and darker due to the abundant buff-colored conidia, while all mutants had the pale or almost colorless colonies of slightly less size and density even two days later (Figure 2C). The liquid culture of the wild type *Th. thermophilus* F-859 strain also grew faster than transgenic strains (Supplementary Figure S1). It can be assumed that fungal energy and resources used up in heterologous protein synthesis negatively affect growth yield of transgenic *Th. thermophilus* rF-859 compared to wild type strain. Although the protein production in the fresh fungal biomass was similar with the control, the dry weights of *TEF*-containing strains were approximately 2-fold higher, probably due to a lower moisture content and, subsequently, a lower weight loss in the mycelium (Figure 2B). It has been previously demonstrated that two fungal species (*Pleurotus ostreatus* and *Trametes multicolor*) grown on varying substrates are characterized by different levels of weight loss after the drying treatment [37]. These parameters are dependent from the fiber types and contribute to the properties of mycelium-based materials [38]. There were no noticeable differences in hyphal morphology between the wild type and rF-859 strains (Figure 2D).

### 3.2. Heterologous Expression of z19 and a1 in Transgenic Strains Th. thermophilus rF-859

**Figure 3:**
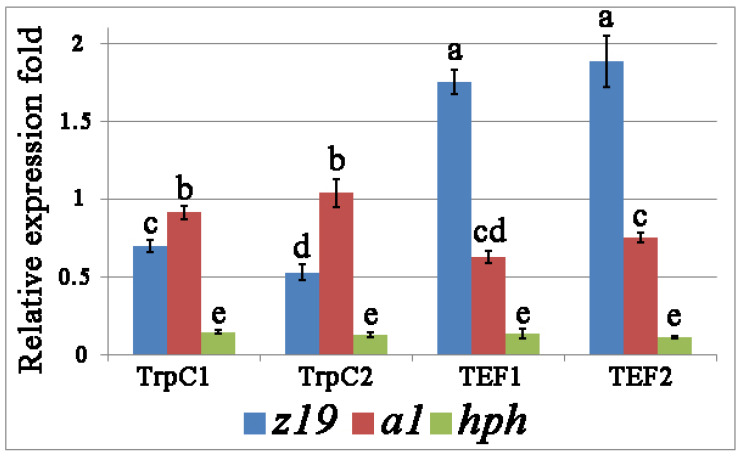
qPCR analysis of *z19*, *a1*, and *hph* expression in *Th. thermophilus* rF-859 strains. Two recombinant clones of *Th. thermophilus* rF-859 harboring the *z19* and *a1* genes driven by P*trpC* (named TrpC1 and TrpC2), as well as two clones expressing the same genes regulated by P*tef* (named TEF1 and TEF2) were analyzed. In all studied clones *hph* gene was driven by P*trpC.* Different letters above the bars indicate statistically significant differences of means (*p* < 0.05) for each of the gene, Fisher’s LSD.

From the quantitative polymerase chain reaction (qPCR) results, the expression levels of *z19* and *a1* in *Th. thermophilus* rF-859 were significantly dependent on their driving promoters, P*tef* or P*trpC* (Figure 3). The P*tef* promoter was 2.3–3.0 times more effective in driving the *z19* transcription than *a1*. On the contrary, the strength of P*trpC* was higher for *a1*, resulting in the nearly 2-fold prevalence of its mRNA abundance against to *z19* in the strain TrpC2 (Figure 3). The *z19* mRNA transcript abundance in the TrpC strains was 2.5–3.7 times lower than that in the TEF strains. The differences in the *a1* expression levels between the genetic constructions was much less evident reaching maximum 1.6-fold elevation in TrpC2 compared with the TEF1 strain. Generally, the patterns of relative expression for the transgenes were characteristic of the TEF or TrpC lineage (Figure 3).

In a previous work [28], we reported that P*trpC* drives the highest level of *hph* expression in the transformed *Th. thermophilus* cells compared to the P*tef* and P*35S* promoters. At the simultaneous expression of three heterologous genes, *hph* under the control of P*trpC* showed the constant but 4 and 7 times lower levels of mRNA compared to *z19* and *a1*, respectively, placed under the same own promoters (Figure 3).

Taken together, our results indicate that the strength of certain promoter is highly variable in the gene-dependent manner. It is probable that the inserted downstream CDS affects significantly the activity of studied promoters and, consequently, the transcription process. It has been postulated that such transcriptional response may be due to the effects on the process of releasing RNA polymerase from the σ subunit [39,40]. The downstream regulatory elements located within CDSs have been previously identified in viruses, yeasts and mammalians [41,42,43]. The gene positional effects were evident during co-expression of fluoresce reporter genes under three heterotypic mammalian synthetic promoters in the positions 2 and 3 that were generally reduced relatively to the position 1 [44]. Moreover, the codon usage may also affect the mRNA levels during heterologous gene expression in *Th. thermophilus*. It has been reported that the codon optimization prevents premature polyadenylation of transcripts, causing an increase in the steady-state mRNA levels of heterologous genes in filamentous fungi [45]. The transgenes *z19*, *a1* and *hph* have the different random codon distribution in their sequences (Supplementary Table S2). However, our analysis revealed that the *hph* sequence contains many fewer numbers of rare codons compared to *z19* and *a1*. The different lifespan, stability and degradation of the heterologously expressed genes may also be taken into consideration.

### 3.3. Accumulation of Plant Storage Proteins in Th. thermophilus rF-859 Strains

**Figure 4:**
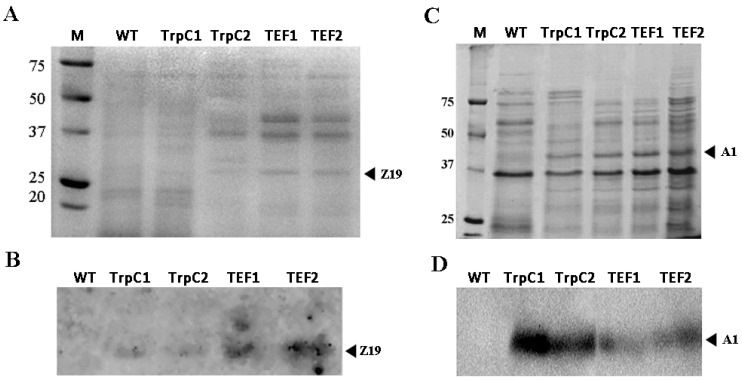
12.5% SDS-PAGE of ethanol-soluble (**A**) and total water-soluble (**C**) protein fractions isolated from the wild type and *Th. thermophilus* rF-859 strains. Detection of Z19 (**B**) and A1 proteins (**D**) using Western blotting with anti-6x-His Tag antibodies. Original Western blots are shown in Supplementary Figure S2.

Among the total proteins isolated from the four recombinant *Th. thermophilus* strains, the presence of maize α-zein Z19 and amaranth albumin A1 in each strain was confirmed by SDS-PAGE and Western blotting analysis (Figure 4, Supplementary Figure S2). Of the four obtained lineages, those expressing the recombinant proteins under the control of P*tef* showed the higher expression level for the ethanol-soluble Z19 (Figure 4A). On the contrary, the accumulation of water-soluble A1 was higher under P*trpC* (Figure 4C).

According to the band intensities, the amount of Z19 varied from 7% to 9% and from 1% to 2% of the ethanol-soluble protein fractions in the TEF and TrpC clones, respectively. The relative amount of the soluble A1 varied from 5% to 6% in the TEF clones (Figure 4A) and from 4% to 6% in the TrpC clones (Figure 4C). The protein identities were confirmed with the use of anti-6x-His Tag antibodies as the bands of interest corresponding to 26 kDa and 39 kDa detected for the Z19 and A1 proteins, respectively (Figure 4B,D, Supplementary Figure S2, Supplementary Table S3). Although the mRNA levels of *z19* were in accordance with the protein Z19 levels, the protein A1 accumulation was approximately comparable in every clone (Figure 3 and Figure 4). This result indicates that the nearly 1.6-fold increase of the *a1* expression levels in the TrpC clones is not sufficient to provide any comparative advantage in the recombinant protein accumulation. On the other hand, only 2-fold *z19* mRNA up-regulation provoked markedly higher product accumulation in the TEF clones. We should also take into consideration that expression of Z19 and A1 may be differently regulated at a post-transcriptional level and the fact that the heterologously expressed α-zein tends to aggregate into insoluble protein bodies, while amaranth albumin remains soluble [11,46]. In this regard, different levels of Z19 and A1 accumulation may be related with protein breakdown under high growth temperature because elevated turnover rate of soluble proteins is considered an important survival strategy of thermophilic fungi [47].

Only exchanging the terminators T*trpC* for T*tef1* for a bidirectional histone-gene promoters system in *Aspergillus* resulted in a significant increase in the relative multigene expression by a factor of 1.8, while they were interchangeable and equally efficient for many other expression constructs [48]. Moreover, the use of the similar promoters in a simultaneously operating dual-promoter vector system was thought to interfere with their effectiveness, because the promoter interference has the potential to occur between vector-borne promoters and endogenous promoters following vector integration [40]. However, the 5′-end of OpIE2 insect viral promoter was found to be blocking the activity of the CMV promoter, constructed in a tandem arrangement, when transforming mammalian cells [49]. Evidently, the promoter interference cannot always be anticipated and empirical validation of functionality should be a prerequisite, as vector containing more than one target gene and promoters and promoter design features can significantly impact performance [40,44].

### 3.4. Amino Acid Profile of Th. thermophilus rF-859

Fungi are able to synthesize all proteinogenic amino acids (AAs), including nine essentials for humans and animals: phenylalanine, valine, threonine, tryptophan, isoleucine, methionine, leucine, lysine, and histidine [50]. The essential and non-essential AAs are not only involved in protein metabolism but also have physiological functions [12]. In agriculture, Met is the first limiting AA in diets in which soybean meal is the main protein, whereas Lys is the first limiting AA when cereal protein predominates [2]. Leucine is generally the second limiting AA. However, Met and Lys are much more influenced by a diet composition than that of Leu, particularly for ruminants because the proteins synthesized by rumen-active microorganisms are deficient in these two amino acids for cattle [2,6]. In most cases, the AA requirements, determined by dose-response studies, are reduced to the diet supplementation of Lys, Met (Met + Cys), Thr, Trp and Val, with increasing availability of their crystalline forms [2,12]. In addition, feeding a reduced crude protein diet with a near ideal AA profile, which can differ during the productive life of animals, improves the AA efficiency and nitrogen utilization [2].

**Figure 5:**
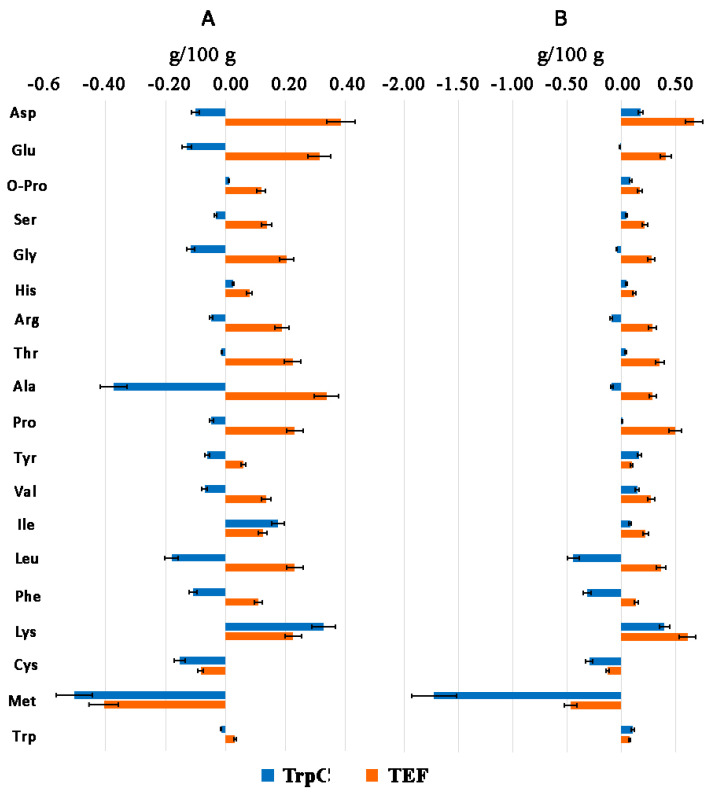
The differences in AA content in *Th. thermophilus* rF-859 clones TrpC (blue) and TEF (orange) relatively to the wild type strain after 14 (**A**) and 28 (**B**) days of cultivation (liquid potato-dextran medium, 45, without shaking).

The analysis of AA composition in *Th. thermophilus* rF-859 (Supplementary Figures S3–S5) during expression of the recombinant genes under the different promoters revealed that the relative total content of every AA is also higher (up to 1.5-fold for Lys) under the P*tef* drive compared to P*trpC*, with the exception of aromatic Tyr and Trp (Figure 5, Table 1). Moreover, Met and Cys were slightly higher in the control (Figure 5, Table 1). In the P*trpC*-containing strains, only Lys, Tyr, Val, Ile, Asp, and Trp were visibly higher than in the control, while the total Leu, Phe, Gly, Arg, Ala, Cys and Met decreased (Supplementary Table S4). These AA ratios remained approximately similar after 28 days of cultivation, but the total AAs decreased by averagely 1.4 times both in the wild type and transgenic strains, particularly by degradation of Lys, Trp, Val, and Pro (Supplementary Tables S4 and S5). Meanwhile, the TEF profile was comparably stable and TrpC became even lower in the most AAs (Figure 5, Supplementary Table S5).

The greatest response to metabolic modification by the P*tef*-driven recombinant proteins was observed in the synthesis of such essential AAs as Lys, Thr, Leu, Val, Ile and Trp (Figure 5, Table 1 and Table 2). Methionine required for all diets was also higher in the P*tef*- vs P*trpC*-containing clones, but slightly reduced in comparison with the control, where Met made the major contribution into the total essential AA content (Figure 5, Table 2). However, *Th. thermophilus* F-859 itself was characterized by approximately 3–6 fold higher Met (64.0) content relative to the next most abundant Ala (27.9), Leu, Asp (14.8), and Val (14.4) (Figure 5, Table 1), probably due to the developed system of *S*-adenosylmethionine(SAM)-dependent methyltransferases and associated methylation processes in their cells in response to a given environmental signal [50,51]. Thus, the high Met level may indicate a high efficacy of sulfur metabolism or/and DNA synthesis apart from initiation of protein translation in *Th. thermophilus* F-859 [50,51]. It is probable that lowering synthesis of a total Met and, consequently, methylation level of DNA at the mycelium stage in transgenic *Th. thermophilus* rF-859 allows its genome to avoid silencing of growth and development processes caused by metabolic overloading due to the recombinant protein production [50,52]. The total Cys also decreases in *Th. thermophilus* rF-859 by the reason of decreasing its precursor Met (Figure 5). The outcomes from labelled AA assay indicated that the highest the Met supply is in diets, the highest is its conversion into Cys [53]. Remarkably, the additional promoter related to the fungal Trp synthesis pathway (*trpC*) also inhibited more largely the host production of the total Met, although its total Trp increased compared to the control (Figure 5A, Table 1).

Thus, the essential AA profiles in the transgenic strains *Th. thermophilus* rF-859 are comparable, but significantly differ by their content of Met+Cys from other filamentous and basidiomycetous fungi, which are grown on low-value by-products and organic-rich waste and are considered the promising sources of feeding supplementation at least in aquaculture (Table 1 and Table 2). Our data show that heterologous overexpression of storage proteins is a new available approach to improve the nutrient value of fungal raw biomass by increasing a protein quality. However, further investigations are needed to achieve a more pronounced effect in regard to the protein yield. The amaranth AmA1-overexpressed sweet potatoes have been reported to be achieved from a 10% to 83% increase in the protein content [11].

Nevertheless, the expression of heterologous protein places a variety of stresses on the host, the most fundamental of which is the added burden of metabolic precursors such as AAs and the energy required for translation of unnecessary proteins, reducing the allocation of cellular resources to growth. Such AAs as His, Trp, and Cys were found to tend to be the most rarely used during normal growth and, therefore, the costliest to biosynthesize in *E. coli* [55]. Given that there are no clear strategies to engineer filamentous fungi for increased secretion of a protein of interest, methods are required that are able to construct and test many different combinations of vectors and promoters, other transcriptional and/or translational control elements, and genome modifications for the high-throughput screening of engineered strains and the correlated scale production [19,44].

## 4. Conclusions

*Th. thermophilus* F-859 can be considered a promising source of indispensable Met, while the transgenic strains *Th. thermophilus* rF-859 provide additionally the improved content of essential Phe, Lys, Val, Tre, Ile, His, and Trp by producing free AAs, as well as the recombinant storage maize α-zein and amaranth albumin A1 of a near ideal AA profile. Although the level of A1 was higher under the control of P*trpc*, the P*tef*-driven constructions of both recombinant proteins contribute more in increasing of essential AAs. Coexpression of α-zein and amaranth albumin could be a useful strategy to boost single-cell protein production in filamentous fungi.

## Figures and Tables

**Table 1:** Amino acid composition of the wild type and transgenic *Th. thermophilus* rF-859 clones TrpC and TEF (* this study, ** AO/FM/SBM—*Aspergillus oryzae*/Fishmeal/Soya bean meal data from the paper by Karimi et al. [17]).

Amino Acids	g/kg DW				% of Total AA Content		
	Wild Type *	TrpC *	TEF *	AO/FM/SBM **	Wild Type *	TrpC *	TEF *
**Essential Amino Acids**							
Histidine	6.7 ± 0.8	7.2 ± 0.9	7.9 ± 0.9	7.51/15.9/11.5	2.63	3.02	2.64
Isoleucine	9.2 ± 1.1	10.0 ± 1.2	11.4 ± 1.4	13.8/25.0/19.9	3.60	4.21	3.82
Leucine	14.8 ± 1.8	10.4 ± 1.2	18.5 ± 2.2	24.6/44.5/31.9	5.82	4.37	6.16
Lysine	12.3 ± 1.5	16.4 ± 2.1	18.5 ± 2.2	21.4/46.4/26.6	4.86	6.88	6.16
Methionine	64.0 ± 7.7	46.7 ± 5.6	59.3 ± 7.1	5.66/16.3/6.2	25.17	19.66	19.80
Phenylalanine	9.2 ± 1.1	6.1 ± 0.7	10.6 ± 1.3	14.2/24.3/21.7	3.60	2.55	3.52
Threonine	10.9 ± 1.4	11.4 ± 1.5	14.5 ± 1.7	16.9/25.6/17.7	4.30	4.78	4.84
Tryptophan	3.2 ± 0.5	4.3 ± 0.7	4.0 ± 0.7	-	1.27	1.82	1.34
Valine	14.4 ± 1.7	15.9 ± 1.9	17.1 ± 2.1	17.2/30.0/20.8	5.66	6.68	5.72
**Non-Essential Amino Acids**							
Arginine	9.9 ± 1.4	9.0 ± 1.3	12.8 ± 1.8	19.9/38.2/32.0	3.88	3.77	4.26
Alanin	27.9 ± 3.3	27.0 ± 3.2	30.8 ± 3.7	23.7/39.7/19.0	10.96	11.36	10.28
Aspartic acid	14.8 ± 1.9	16.6 ± 2.2	21.5 ± 2.8	-	5.82	6.99	7.19
Cystein	8.8 ± 1.1	5.8 ± 0.8	7.6 ± 1.0	2.75/5.1/0.5	3.45	2.46	2.53
Glutamic acid	9.5 ± 1.1	9.4 ± 1.1	13.6 ± 1.6	43.3/77.3/77.0	3.75	3.96	4.55
Glycin	7.7 ± 0.9	7.4 ± 0.9	10.6 ± 1.3	17.2/48.0/18.1	3.05	3.10	3.52
Hydroxyprolin	5.3 ± 1.0	6.2 ± 1.1	7.0 ± 1.3	-	2.08	2.60	2.35
Proline	13.1 ± 1.6	13.2 ± 1.6	18.0 ± 2.2	18.5/28.3/21.6	5.13	5.53	6.02
Serine	7.0 ± 0.8	7.5 ± 0.9	9.2 ± 1.1	17.2/24.5/21.8	2.77	3.18	3.08
Tyrosine	5.6 ± 0.7	7.3 ± 0.9	6.6 ± 0.8	12.8/19.0/14.6	2.21	3.07	2.20
Total amino acids	254.2	237.8	299.5	168.7/299.6/221.6	100	100	100

**Table 2:** Calculated nutritional quality of the wild type and transgenic *Th. thermophilus* rF-859 clones TrpC and TEF (* Nutritional quality was calculated as described by Lata and Atri [54]).

Nutritional Quality *	Results		
	Wild Type	TrpC	TEF
Total Amino Acid (TAA), %	25.42	23.78	29.95
Total Essential Amino Acid/Total Non-Essential Amino Acid	1.32	1.17	1.17
Total Sulphur Amino Acid (TSAA = Meth + Cys), %	7.28	5.25	6.69
Total Aromatic Essential Amino Acid (TArEAA = Phe + Tyr), %	1.48	1.37	1.72
Total Essential Amino Acids, %	56.91	53.97	54.00
Total Non-Essential Amino Acid (TNEAA)/Total Amino Acid (TAA)	43.10	46.02	45.98

## Data Availability

All data are contained within the article and Supplementary Materials.

## Supplementary Materials

The following supporting information can be downloaded at https://www.mdpi.com/article/10.3390/jof8020119/s1, Table S1: The total protein content of extracts from the wild type (WT) and *Th. thermophilus* rF-859 strains; Table S2: Triplet frequencies for the heterologously expressed *z19*, *a1*, and *hph* genes in comparison with codon usage of *Aspergillus niger*. * Start (ATG) and stop (TAA, TAG, TGA) codons are omitted; Table S3: The AA composition calculated from the sequences of the mature storage proteins: maize α-zein B1 (Genbank acc. no. AF371269) and amaranth albumin A1 (Genbank acc. no. AF491291). * Acidic = 3 Basic = 14, Non-Polar = 139, Polar = 94, total AA = 233, MW = 25,309.3; ** Acidic = 38, Basic = 48, Non-Polar = 136, Polar = 167, total AA = 303, MW = 34,831.0 (Gene Runner 6.0, https://gene-runner.software.informer.com/6.0/ accessed on 23rd January 2022); Table S4: Relative amino acid content in the wild type *Th. thermophilus* F-859 and transgenic *Th. thermophilus* rF-859 clones TrpC and TEF. * DW—dry weight; Table S5: Change of the AA content in the wild type *Th. thermophilus* F-859 and transgenic *Th. thermophilus* rF-859 clones TrpC and TEF after 14 days of growth relatively to the AA content after 28 days of growth (AA 14 /AA 28 days). Figure S1: Time course of fresh (A) and dry (B) weight accumulation for the wild type (WT) and *Th. thermophilus* rF-859 strains; Figure S2: Original Western blot data for Figure 4; Figure S3: HPLC chromatogram (LC-20 Prominence, Shimadzu, Japan) for determination of Met and Cys; Figure S4: HPLC chromatogram (LC-20 Prominence, Shimadzu, Japan) for determination of Asp, Glu, o-Pro, Ser, Gly, His, Arg, Thr, Ala, Pro, Tyr, Val, Ile, Leu, Phe, Trp, Lys; Figure S5. HPLC chromatogram (LC-20 Prominence, Shimadzu, Japan) for determination of Trp, and Supplementary Materials and Methods sections.

## References

[1] Parisi G., Tulli F., Fortina R., Marino R., Bani P., Dalle Zotte A., De Angelis A., Piccolo G., Pinotti L., Schiavone A. (2020). Protein Hunger of the Feed Sector: The Alternatives Offered by the Plant World. Ital. J. Anim. Sci..

[2] Zhang S., Qiao M., Trottier N.L. (2019). Feeding a Reduced Protein Diet with a near Ideal Amino Acid Profile Improves Amino Acid Efficiency and Nitrogen Utilization for Milk Production in Sows. J. Anim. Sci..

[3] Dhillon G.S., Dhillon G.S. (2016). Protein Byproducts. Transformation from Environmental Burden into Value-Added Products.

[4] Li M.H., Robinson E.H., Bosworth B.G., Oberle D.F., Lucas P.M. (2011). Use of Corn Gluten Feed and Cottonseed Meal to Replace Soybean Meal and Corn in Diets for Pond-Raised Channel Catfish. N. Am. J. Aquac..

[5] Schweiggert-Weisz U., Eisner P., Bader-Mittermaier S., Osen R. (2020). Food Proteins from Plants and Fungi. Curr. Opin. Food Sci..

[6] Loy D.D., Lundy E.L. (2019). Chapter 23—Nutritional Properties and Feeding Value of Corn and Its Coproducts. Corn.

[7] Gibbon B.C., Larkins B.A. (2005). Molecular Genetic Approaches to Developing Quality Protein Maize. Trends Genet..

[8] Sánchez-López F., Robles-Olvera V.J., Hidalgo-Morales M., Tsopmo A. (2020). Characterization of *Amaranthus hypochondriacus* Seed Protein Fractions, and Their Antioxidant Activity after Hydrolysis with Lactic Acid Bacteria. J. Cereal Sci..

[9] Manyelo T.G., Sebola N.A., van Rensburg E.J., Mabelebele M. (2020). The Probable Use of Genus *Amaranthus* as Feed Material for Monogastric Animals. Animals.

[10] Chakraborty S., Chakraborty N., Datta A. (2000). Increased Nutritive Value of Transgenic Potato by Expressing a Nonallergenic Seed Albumin Gene from *Amaranthus hypochondriacus*. Proc. Natl. Acad. Sci. USA.

[11] Shekhar S., Agrawal L., Mishra D., Buragohain A.K., Unnikrishnan M., Mohan C., Chakraborty S., Chakraborty N. (2016). Ectopic Expression of Amaranth Seed Storage Albumin Modulates Photoassimilate Transport and Nutrient Acquisition in Sweetpotato. Sci. Rep..

[12] van Milgen J., Dourmad J.-Y. (2015). Concept and Application of Ideal Protein for Pigs. J. Anim. Sci. Biotechnol..

[13] Sari Y.W. (2015). Biomass and Its Potential for Protein and Amino Acids: Valorizing Agricultural By-Products.

[14] Sarnklong C., Cone J., Pellikaan W.F., Hendriks W. (2010). Utilization of Rice Straw and Different Treatments to Improve Its Feed Value for Ruminants: A Review. Anim. Biosci..

[15] Ritala A., Häkkinen S.T., Toivari M., Wiebe M.G. (2017). Single Cell Protein—State-of-the-Art, Industrial Landscape and Patents 2001–2016. Front. Microbiol..

[16] Ntana F., Mortensen U.H., Sarazin C., Figge R. (2020). Aspergillus: A Powerful Protein Production Platform. Catalysts.

[17] Karimi S., Mahboobi Soofiani N., Lundh T., Mahboubi A., Kiessling A., Taherzadeh M.J. (2019). Evaluation of Filamentous Fungal Biomass Cultivated on Vinasse as an Alternative Nutrient Source of Fish Feed: Protein, Lipid, and Mineral Composition. Fermentation.

[18] Baker S.E. (2018). Protein Hyperproduction in Fungi by Design. Appl. Microbiol. Biotechnol..

[19] Arnau J., Yaver D., Hjort C.M., Nevalainen H. (2020). Strategies and Challenges for the Development of Industrial Enzymes Using Fungal Cell Factories. Grand Challenges in Fungal Biotechnology.

[20] Wang Q., Zhong C., Xiao H. (2020). Genetic Engineering of Filamentous Fungi for Efficient Protein Expression and Secretion. Front. Bioeng. Biotechnol..

[21] Brancoli P., Gmoser R., Taherzadeh M.J., Bolton K. (2021). The Use of Life Cycle Assessment in the Support of the Development of Fungal Food Products from Surplus Bread. Fermentation.

[22] Xu J., Li J., Lin L., Liu Q., Sun W., Huang B., Tian C. (2015). Development of Genetic Tools for *Myceliophthora thermophila*. BMC Biotechnol..

[23] Liu Q., Gao R., Li J., Lin L., Zhao J., Sun W., Tian C. (2017). Development of a Genome-Editing CRISPR/Cas9 System in Thermophilic Fungal *Myceliophthora* Species and Its Application to Hyper-Cellulase Production Strain Engineering. Biotechnol. Biofuels.

[24] Li J., Zhang Y., Li J., Sun T., Tian C. (2020). Metabolic Engineering of the Cellulolytic Thermophilic Fungus *Myceliophthora thermophila* to Produce Ethanol from Cellobiose. Biotechnol. Biofuels.

[25] Kern A., Shanahan D., Buesen R., Geiger D. (2020). Safety Evaluation of a β-Mannanase Enzyme Preparation Produced with *Thermothelomyces thermophilus* Expressing a Protein-Engineered β-Mannanase Gene. PLoS ONE.

[26] Visser H., Joosten V., Punt P.J., Gusakov A.V., Olson P.T., Joosten R., Bartels J., Visser J., Sinitsyn A.P., Emalfarb M.A. (2011). Development of a Mature Fungal Technology and Production Platform for Industrial Enzymes Based on a *Myceliophthora thermophila* Isolate, Previously Known as *Chrysosporium lucknowense* C1. Ind. Biotechnol..

[27] Shkryl Y.N., Veremeichik G.N., Makhazen D.S., Silantieva S.A., Mishchenko N.P., Vasileva E.A., Fedoreyev S.A., Bulgakov V.P. (2016). Increase of Anthraquinone Content in *Rubia cordifolia* Cells Transformed by Native and Constitutively Active Forms of the *AtCPK1* Gene. Plant Cell Rep..

[28] Balabanova L.A., Shkryl Y.N., Slepchenko L.V., Yugay Y.A., Gorpenchenko T.Y., Kirichuk N.N., Khudyakova Y.V., Bakunina I.Y., Podvolotskaya A.B., Bulgakov V.P. (2019). Development of Host Strains and Vector System for an Efficient Genetic Transformation of Filamentous Fungi. Plasmid.

[29] Tzfira T., Tian G.-W., Lacroix B., Vyas S., Li J., Leitner-Dagan Y., Krichevsky A., Taylor T., Vainstein A., Citovsky V. (2005). PSAT Vectors: A Modular Series of Plasmids for Autofluorescent Protein Tagging and Expression of Multiple Genes in Plants. Plant Mol. Biol..

[30] Bulgakov V.P., Veremeichik G.N., Shkryl Y.N. (2015). The *rolB* Gene Activates the Expression of Genes Encoding MicroRNA Processing Machinery. Biotechnol. Lett..

[31] Hood E.E., Gelvin S.B., Melchers L.S., Hoekema A. (1993). New *Agrobacterium* Helper Plasmids for Gene Transfer to Plants. Transgenic Res..

[32] Bradford M.M. (1976). A Rapid and Sensitive Method for the Quantitation of Microgram Quantities of Protein Utilizing the Principle of Protein-Dye Binding. Anal. Biochem..

[33] Fic E., Kedracka-Krok S., Jankowska U., Pirog A., Dziedzicka-Wasylewska M. (2010). Comparison of Protein Precipitation Methods for Various Rat Brain Structures Prior to Proteomic Analysis. Electrophoresis.

[34] Woo Y.-M., Hu D.W.-N., Larkins B.A., Jung R. (2001). Genomics Analysis of Genes Expressed in Maize Endosperm Identifies Novel Seed Proteins and Clarifies Patterns of Zein Gene Expression. Plant Cell.

[35] Pisarikova B., Kracmar S., Herzig I. (2005). Amino Acid Contents and Biological Value of Protein in Various Amaranth Species. Czech J. Anim. Sci..

[36] Rantasalo A., Landowski C.P., Kuivanen J., Korppoo A., Reuter L., Koivistoinen O., Valkonen M., Penttilä M., Jäntti J., Mojzita D. (2018). A Universal Gene Expression System for Fungi. Nucleic Acids Res..

[37] Appels F.V.W., Camere S., Montalti M., Karana E., Jansen K.M.B., Dijksterhuis J., Krijgsheld P., Wöstena H.A.B. (2019). Fabrication Factors Influencing Mechanical, Moisture- and Water-Related Properties of Mycelium-Based Composites. Mater. Des..

[38] Elsacker E., Vandelook S., Brancart J., Peeters E., De Laet L. (2019). Mechanical, Physical and Chemical Characterisation of Mycelium-Based Composites with Different Types of Lignocellulosic Substrates. PLoS ONE.

[39] Tran T.T., Charles T.C. (2020). The Variation of Promoter Strength in Different Gene Contexts. bioRxiv.

[40] Duzenli O.F., Okay S., Duzenli O.F., Okay S. (2020). Promoter Engineering for the Recombinant Protein Production in Prokaryotic Systems. AIMS Bioeng..

[41] He X., Fütterer J., Hohn T. (2002). Contribution of Downstream Promoter Elements to Transcriptional Regulation of the Rice Tungro Bacilliform Virus Promoter. Nucleic Acids Res..

[42] Wenz P., Schwank S., Hoja U., Schüller H.J. (2001). A Downstream Regulatory Element Located within the Coding Sequence Mediates Autoregulated Expression of the Yeast Fatty Acid Synthase Gene *FAS2* by the *FAS1* Gene Product. Nucleic Acids Res..

[43] Lee N., Iyer S.S., Mu J., Weissman J.D., Ohali A., Howcroft T.K., Lewis B.A., Singer D.S. (2010). Three Novel Downstream Promoter Elements Regulate MHC Class I Promoter Activity in Mammalian Cells. PLoS ONE.

[44] Patel Y.D., Brown A.J., Zhu J., Rosignoli G., Gibson S.J., Hatton D., James D.C. (2021). Control of Multigene Expression Stoichiometry in Mammalian Cells Using Synthetic Promoters. ACS Synth. Biol..

[45] Tokuoka M., Tanaka M., Ono K., Takagi S., Shintani T., Gomi K. (2008). Codon Optimization Increases Steady-State MRNA Levels in *Aspergillus oryzae* Heterologous Gene Expression. Appl. Environ. Microbiol..

[46] Coleman C.E., Herman E.M., Takasaki K., Larkins B.A. (1996). The Maize Gamma-Zein Sequesters Alpha-Zein and Stabilizes Its Accumulation in Protein Bodies of Transgenic Tobacco Endosperm. Plant Cell.

[47] Maheshwari R., Bharadwaj G., Bhat M.K. (2000). Thermophilic Fungi: Their Physiology and Enzymes. Microbiol. Mol. Biol. Rev..

[48] Rendsvig J.K.H., Workman C.T., Hoof J.B. (2019). Bidirectional Histone-Gene Promoters in *Aspergillus*: Characterization and Application for Multi-Gene Expression. Fungal Biol. Biotechnol..

[49] Aladdin A., Sahly N., Faty R., Youssef M.M., Salem T.Z. (2021). The Baculovirus Promoter OpIE2 Sequence Has Inhibitory Effect on the Activity of the Cytomegalovirus (CMV) Promoter in HeLa and HEK-293 T Cells. Gene.

[50] Jastrzębowska K., Gabriel I. (2015). Inhibitors of Amino Acids Biosynthesis as Antifungal Agents. Amino Acids.

[51] Bischof R.H., Horejs J., Metz B., Gamauf C., Kubicek C.P., Seiboth B. (2015). L-Methionine Repressible Promoters for Tuneable Gene Expression in *Trichoderma reesei*. Microb. Cell Fact..

[52] Nai Y.-S., Huang Y.-C., Yen M.-R., Chen P.-Y. (2021). Diversity of Fungal DNA Methyltransferases and Their Association with DNA Methylation Patterns. Front. Microbiol..

[53] Pacheco L.G., Sakomura N.K., Suzuki R.M., Dorigam J.C.P., Viana G.S., Van Milgen J., Denadai J.C. (2018). Methionine to Cystine Ratio in the Total Sulfur Amino Acid Requirements and Sulfur Amino Acid Metabolism Using Labelled Amino Acid Approach for Broilers. BMC Vet. Res..

[54] Atri N.S. (2017). Amino Acid Profile of a Basidiomycetous Edible Mushroom—*Lentinus sajor-caju*. Int. J. Pharm. Pharm. Sci..

[55] Tan J., Sastry A.V., Fremming K.S., Bjørn S.P., Hoffmeyer A., Seo S., Voldborg B.G., Palsson B.O. (2020). Independent Component Analysis of *E. Coli*’s Transcriptome Reveals the Cellular Processes That Respond to Heterologous Gene Expression. Metab. Eng..

