# Supplementary material for: Engineered Fungus Thermothelomyces thermophilus Producing Plant Storage Proteins
Source: J Fungi (Basel). 2022 Jan 26;8(2):119. doi: 10.3390/jof8020119 (PMC8877005; doi:10.3390/jof8020119)
Supplement: Supplementary file 1 [file jof-08-00119-s001.zip › jof-1544937-supplementary.pdf]

Supplementary Table S1. The total protein content of extracts from the wild type (WT) and *Th. thermophilus* rF-859 strains.

Strain	Protein content, mg/ml
WT	0.521±0.063
TrpC1	0.517±0.055
TrpC2	0.534±0.066
Tef1	0.537±0.068
Tef2	0.528±0.063

Supplementary Table S2. Triplet frequencies for the heterologously expressed *z19*, *a1*, and *hph* genes in comparison with codon usage of *Aspergillus niger*. *Start (ATG) and stop (TAA, TAG, TGA) codons are omitted.

AAs	Codon*	%, frequency per one hundred triplets			
		z19	*a1*	*hph*	*A. niger* (13905 CDSs)
Ala	GCG	1.3	0.7	0.4	1.7
	GCA	3.8	1.0	1.8	1.7
	GCT	6.8	2.3	2.1	2.2
	GCC	2.1	0.7	2.9	2.7
Cys	TGT	0.4	0.7	0.6	0.6
	TGC	0.9	1.0	1.5	0.8
Asp	GAT	0.0	4.9	4.1	2.8
	GAC	0.0	0.3	3.5	2.7
Glu	GAG	1.0	1.0	3.2	3.5
	GAA	3.9	3.9	3.2	2.5
Phe	TTT	4.6	4.6	0.6	1.3
	TTC	1.0	1.0	4.1	2.4
Gly	GGG	1.3	1.3	1.8	1.3
	GGA	1.3	1.3	2.0	1.3
	GGT	2.3	2.3	1.5	1.8
	GGC	0.3	0.3	1.8	2.3
His	CAT	1.6	1.6	0.6	1.2
	CAC	0.7	0.7	1.2	1.3
Ile	ATA	1.6	1.6	0.6	0.7
	ATT	3.9	3.9	1.5	1.7
	ATC	1.0	1.0	2.0	2.3
Lys	AAG	0.0	2.3	1.2	3.0
	AAA	0.4	4.3	0.6	1.4
Leu	TTG	4.7	1.6	0.9	1.6
	TTA	3.0	3.3	0.0	0.5
	CTG	1.3	0.0	2.6	2.3
	CTA	3.8	0.7	0.0	0.9
	CTT	4.7	2.6	1.5	1.5
	CTC	2.6	0.3	2.6	2.2
Asn	AAT	0.0	6.6	1.2	1.5
	AAC	4.3	2.6	0.9	2.1
Pro	CCG	0.9	0.0	2.3	1.4
	CCA	4.3	2.0	0.3	1.3
	CCT	2.6	1.0	0.6	1.5
	CCC	2.1	0.7	1.2	1.8
Gln	CAG	4.7	0.3	2.6	2.4

	CAA	1.3	4.3	2.0	1.6
Arg	AGG	0.9	0.7	0.9	0.8
	AGA	0.0	1.0	0.3	0.8
	CGG	0.0	0.0	1.8	1.1
	CGA	0.0	0.3	0.9	0.9
	CGT	0.0	0.0	2.0	1.0
	CGC	0.0	0.7	1.8	1.6
Ser	AGT	0.4	1.0	0.6	1.1
	AGC	1.3	0.3	3.2	1.5
	TCG	0.4	0.7	0.9	1.4
	TCA	2.1	2.0	0.0	1.1
	TCT	3.0	1.6	0.6	1.4
	TCC	0.4	1.3	1.8	1.9
Thr	ACG	0.4	0.7	1.5	1.3
	ACA	1.7	2.0	0.9	1.2
	ACT	0.0	2.6	1.2	1.4
	ACC	1.3	0.7	1.5	2.1
Val	GTG	1.7	1.3	1.2	1.9
	GTA	0.4	1.0	1.2	0.7
	GTT	0.0	2.0	0.9	1.5
	GTC	0.0	2.0	4.1	2.2
Trp	TGG	0.0	2.3	2.3	1.5
Tyr	TAT	1.3	3.6	2.0	1.2
	TAC	0.2	1.3	1.2	1.7

Supplementary Table S3. The AA composition calculated from the sequences of the mature storage proteins: maize *α*-zein B1 (Genbank acc. no. AF371269) and amaranth albumin A1 (Genbank acc. no. AF491291). *Acidic = 3 Basic = 14, Non-Polar = 139, Polar = 94, total AA = 233, MW = 25309.3; **Acidic = 38, Basic = 48, Non-Polar = 136, Polar = 167, total AA = 303, MW = 34831.0 (Gene Runner 6.0, https://gene-runner.software.informer.com/6.0/ accessed on 23rd January 2022).

**Amino acids (%)**	***α*-zein B1***	**A1****
Histidine (H)	0.85	2.31
Isoleucine (I)	4.29	6.60
Leucine (L)	20.17	8.58
Lysine (K)	0.43	6.60
Methionine (M)	0.43	1.98
Phenylalanine (F)	6.01	5.61
Threonine (T)	3.43	5.94
Tryptophan (W)	0	2.31
Valine (V)	2.15	6.25
Arginine (R)	0.86	2.64
Alanin (A)	14.16	4.62
Aspartic acid (D)	0	5.28
Cystein (C)	1.29	1.65

Glutamic acid (E)	0.43	4.95
Glycin (G)	2.58	5.28
Proline (P)	9.83	3.63
Serine (S)	7.73	6.93
Tyrosine (Y)	3.43	4.93
Asparagine (N)	4.29	9.24
Glutamine (Q)	17.60	4.62

Supplementary Table S4. Relative amino acid content in the wild type *Th. thermophilus* F-859 and transgenic *Th. thermophilus* rF-859 clones TrpC and TEF. *DW - dry weight.

Amino Acids	g/100g DW* (14 days)			g/100g DW (28 days)		
	**Wild type** (WT)	**TrpC** (TrpC/WT)	**TEF** (TEF/WT)	**Wild type**	**TrpC** (TrpC/WT)	**TEF** (TEF/WT)
**Essential Amino Acids**						
Histidine	0.67±0.08	0.72±0.09 (1.07)	0.79±0.09 (1.17)	0.44±0.05	0.47±0.06 (1.07)	0.52±0.06 (1.18)
Isoleucine	0.92±0.11	1.00±0.12 (1.1)	1.14±0.14 (1.24)	0.50±0.06	0.68±0.08 (1.36)	0.63±0.08 (1.26)
Leucine	1.48±0.18	1.04±0.12 (0.7)	1.85±0.22 (1.25)	0.93±0.11	0.75±0.09 (0.8)	1.16±0.14 (1.25)
Lysine	1.23±0.15	1.64±0.21 (1.33)	1.85±0.22 (1.5)	0.46±0.05	0.79±0.09 (1.7)	0.68±0.08 (1.48)
Methionine	6.40±0.77	4.67±0.56 (0.73)	5.93±0.71 (0.92)	5.56±0.67	5.06±0.61 (0.91)	5.16±0.62 (0.92)
Phenylalanine	0.92±0.11	0.61±0.07 (0.66)	1.06±0.13 (1.15)	0.73±0.09	0.62±0.07 (0.85)	0.84±0.10 (1.15)
Threonine	1.09±0.14	1.14±0.15 (1.05)	1.45±0.17 (1.33)	0.69±0.09	0.67±0.09 (0.97)	0.91±0.12 (1.32)
Tryptophan	0.32±0.05	0.43±0.07 (1.34)	0.40±0.07 (1.25)	0.13±0.02	0.12±0.02 (0.92)	0.16±0.03 (1.23)
Valine	1.44±0.17	1.59±0.19 (1.1)	1.71±0.21 (1.19)	0.71±0.08	0.64±0.08 (0.9)	0.84±0.10 (1.18)
**Non-Essential Amino Acids**						
Arginine	0.99±0.14	0.90±0.13 (0.9)	1.28±0.18 (1.29)	0.65±0.09	0.60±0.08 (0.92)	0.84±0.12 (1.29)
Alanin	2.79±0.33	2.70±0.32 (0.97)	3.08±0.37 (1.1)	3.22±0.39	2.85±0.34 (0.89)	3.56±0.43 (1.1)
Aspartic acid	1.48±0.19	1.66±0.22 (1.12)	2.15±0.28 (1.45)	0.85±0.11	0.75±0.01 (0.88)	1.24±0.16 (1.46)
Cystein	0.88±0.11	0.58±0.08 (0.66)	0.76±0.10 (0.86)	0.60±0.08	0.45±0.06 (0.75)	0.52±0.07 (0.87)
Glutamic acid	0.95±0.11	0.94±0.11 (0.99)	1.36±0.16 (1.43)	0.73±0.09	0.60±0.07 (0.82)	1.04±0.13 (1.42)
Glycin	0.77±0.09	0.74±0.09 (0.96)	1.06±0.13 (1.38)	0.57±0.07	0.45±0.05 (0.79)	0.77±0.09 (1.35)

Hydroxyprolin	0.53±0.10	0.62±0.11 (1.17)	0.70±0.13 (1.32)	0.36±0.07	0.37±0.07 (1.02)	0.48±0.09 (1.33)
Proline	1.31±0.16	1.32±0.16 (1.00)	1.80±0.22 (1.37)	0.61±0.07	0.56±0.07 (0.92)	0.84±0.10 (1.38)
Serine	0.70±0.08	0.75±0.09 (1.07)	0.92±0.11 (1.31)	0.44±0.05	0.41±0.05 (0.93)	0.58±0.07 (1.32)
Tyrosine	0.56±0.07	0.73±0.09 (1.3)	0.66±0.08 (1.18)	0.34±0.04	0.28±0.03 (0.82)	0.40±0.05 (1.18)
Total amino acids	25.42	23.78 (0.94)	29.95 (1.18)	18.52	17.12 (0.93)	21.18 (1.14)

Supplementary Table S5. Change of the AA content in the wild type *Th. thermophilus* F-859 and transgenic *Th. thermophilus* rF-859 clones TrpC and TEF after 14 days of growth relatively to the AA content after 28 days of growth (AA 14 /AA 28 days).

**Amino Acids**	**Wild type**	**TrpC**	**TEF**
**Essential Amino Acids**			
Histidine	1.52	1.53	1.51
Isoleucine	1.84	1.47	1.80
Leucine	1.59	1.38	1.59
Lysine	2.67	2.07	2.72
Methionine	1.15	0.92	1.14
Phenylalanine	1.26	0.98	1.26
Threonine	1.57	1.70	1.59
Tryptophan	2.46	3.58	2.5
Valine	2.02	2.48	2.03
**Non-Essential Amino Acids**			
Arginine	1.52	1.5	1.52
Alanin	0.86	0.94	0.86
Aspartic acid	1.74	2.21	1.73
Cystein	1.46	1.28	1.46
Glutamic acid	1.30	1.56	1.30
Glycin	1.35	1.64	1.37
Hydroxyprolin	1.47	1.67	1.45
Proline	2.14	2.35	2.14
Serine	1.59	1.82	1.58
Tyrosine	1.64	2.6	1.65
Total amino acids	1.37	1.38	1.41

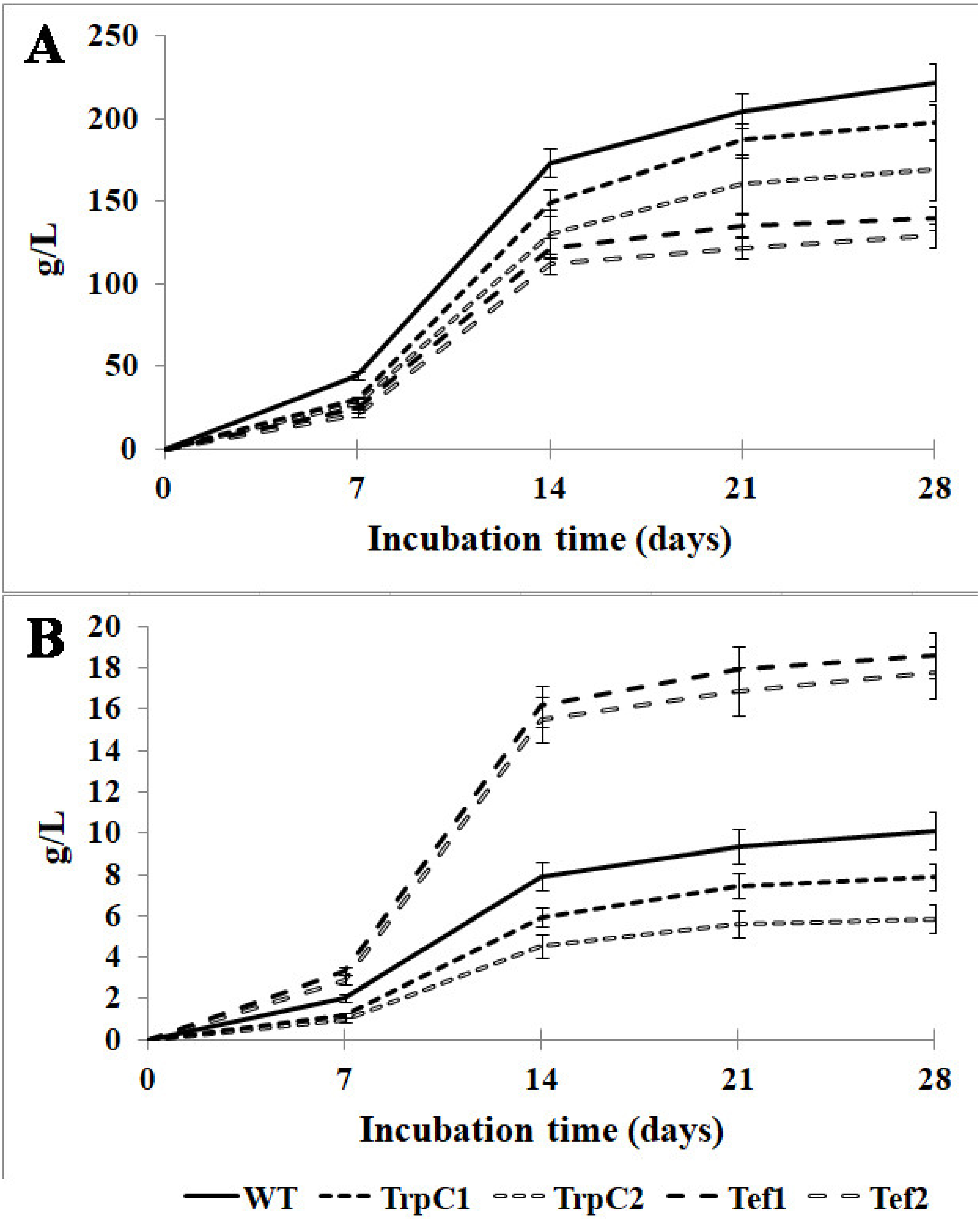

Supplementary Figure S1. Time course of fresh (**A**) and dry (**B**) weight accumulation for the wild type (WT) and *Th. thermophilus* rF-859 strains (in g/L). Values and error bars represent means and standard errors of independent triplicate experiments.

Z19 A1

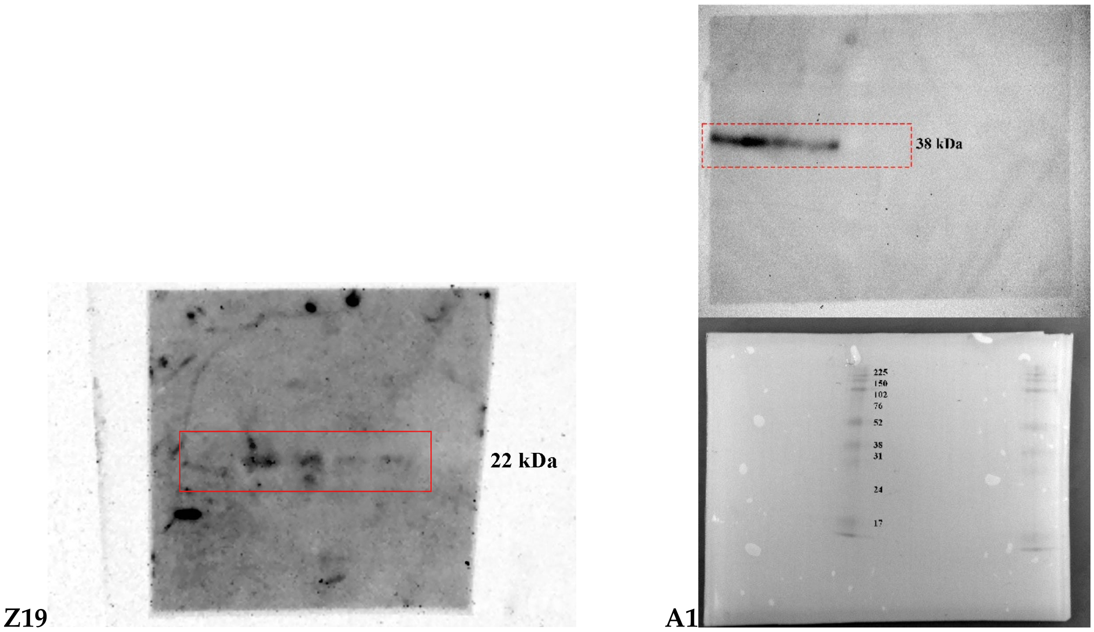

Supplementary Figure S2. Original western blot data for Fig. 4 B and D.

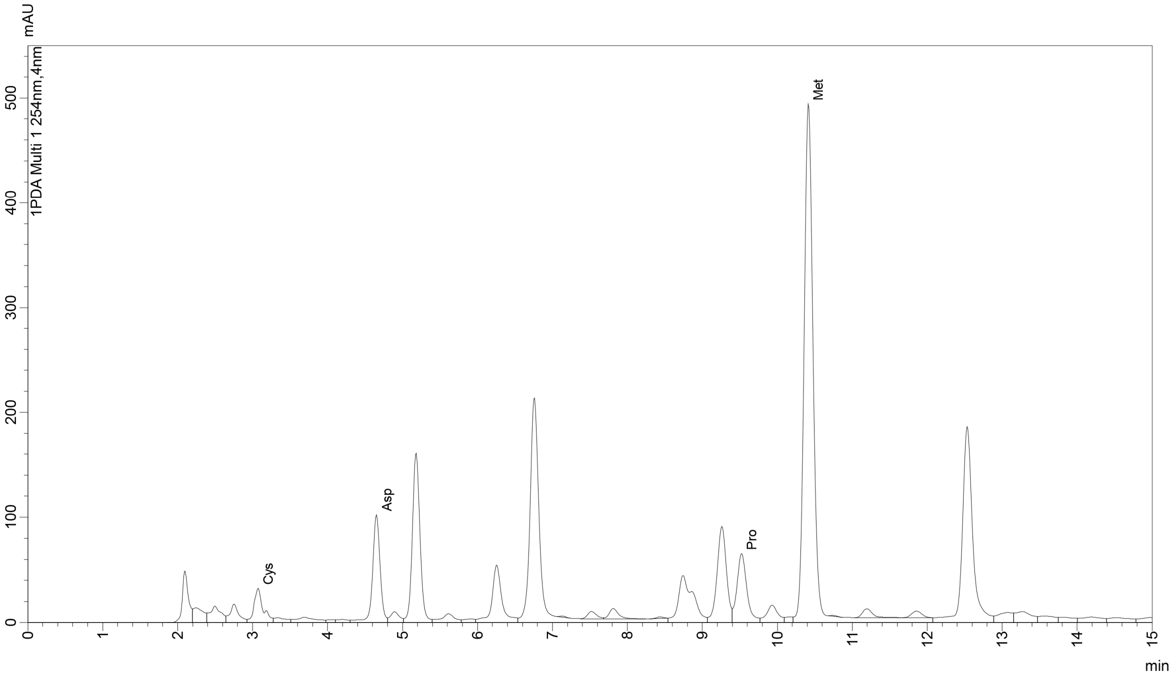

Supplementary Figure S3. HPLC chromatogram (LC-20 Prominence, Shimadzu, Kyoto, Japan) for determination of Met and Cys.

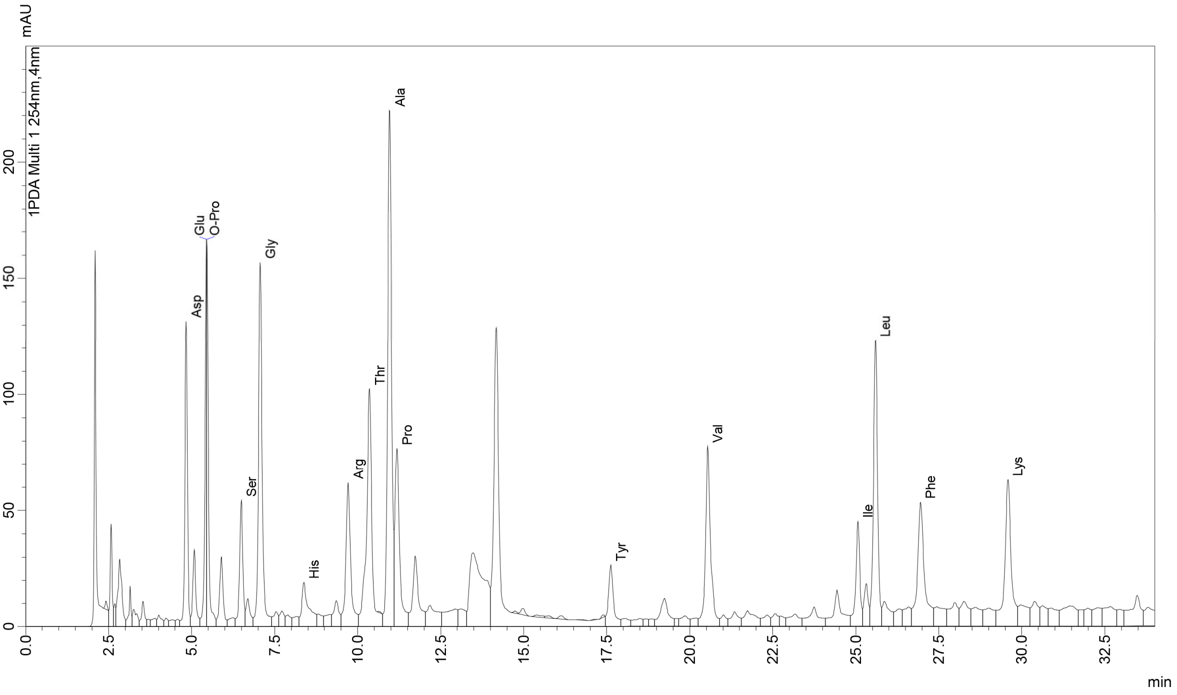

Supplementary Figure S4. HPLC chromatogram (LC-20 Prominence, Shimadzu, Kyoto, Japan) for determination of Asp, Glu, o-Pro, Ser, Gly, His, Arg, Thr, Ala, Pro, Tyr, Val, Ile, Leu, Phe, Trp, Lys.

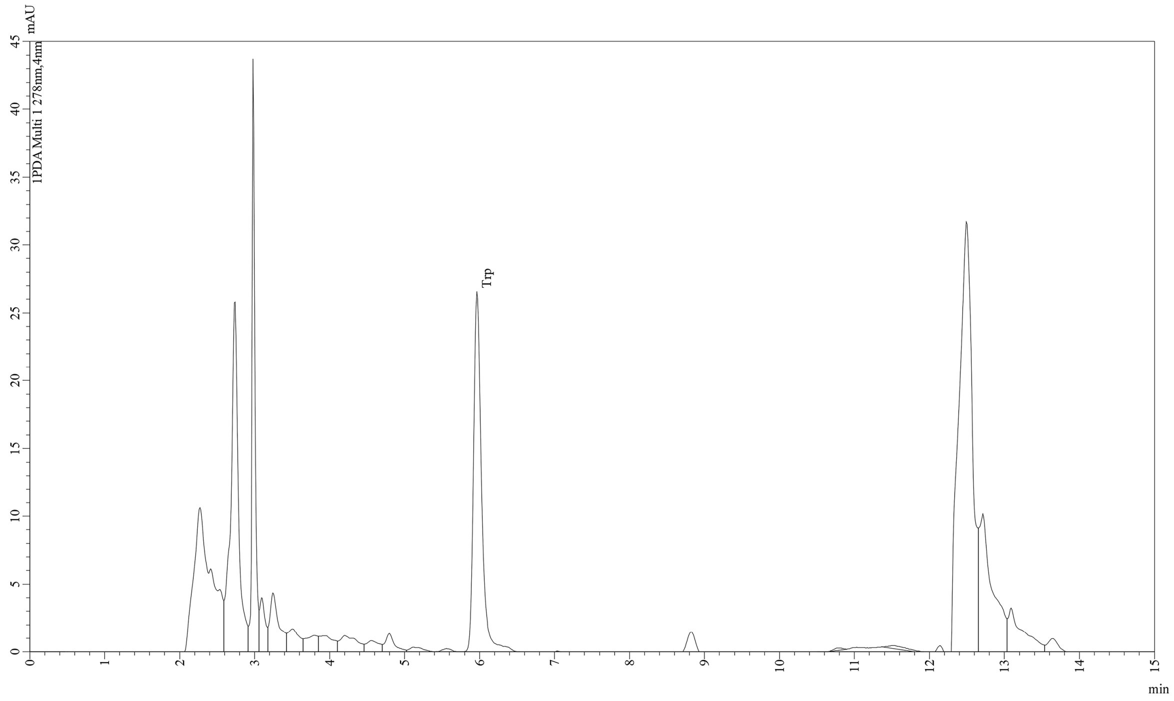

Supplementary Figure S5. HPLC chromatogram (LC-20 Prominence, Shimadzu, Kyoto, Japan) for determination of Trp.

Supplementary Materials and Methods.

Mitotic stability analysis. To evaluate mitotic stability of the transgenic *Th. thermophilus* rF-859 clones TrpC and TEF, twenty-five randomly selected transformants were cultured on PDA plates without antibiotic for 7 days. Mycelium from the edges of the cultures was transferred to the fresh PDA plates and grown for another seven days. After repeating this procedure for five times, germinating mycelia from each transformant were transferred to PDA plates containing 12.5 μg/ml hygromycin and checked for the presence of hph gene as described above.

Extraction of Z19. Ethanol-soluble proteins were extracted from 14-days old mycelia of *Th. thermophilus* using 10 volumes of 60% 2-propanol with 0.6% β-mercaptoethanol. Three rounds of ultrasonic homogenization at 40% output with 2 minutes intervals on ice were performed using ultrasonic homogenizer SONOPULS HD 2070 (BANDELIN electronic GmbH & Co. KG, Berlin, Germany). Afterwards homogenates were left overnight at room temperature with constant stirring at 200 rpm. The next day cell debris was removed by centrifugation at 10,000 g for 30 minutes. The volume of resulting supernatants was reduced using SpeedVac vacuum concentrator (Eppendorf, Hamburg, Germany) and proteins were precipitated with water followed by centrifugation. The protein pellet recovered after centrifugation was dissolved in 2 M urea with 0.1% SDS prior protein concentration measurement.

Extraction of A1 protein. Soluble proteins were extracted from 14-days old mycelia using 10 volumes of solubilization buffer containing 50 mM K-phosphate buffer, pH 8.0, 400 mM NaCl, 20% glycerol, and 5% β-mercaptoethanol. Homogenates were sonicated three times at 40% output with 2 minutes rest on ice and left overnight with constant stirring at 4°C. Cell debris was removed by centrifugation at 18 000 g for 20 minutes at +4 °C. Proteins were concentrated with chlorophorm/methanol as described (Fic et al., 2010). Pellets were dried and dissolved in 2 M urea and 0.1% SDS.

SDS-PAGE. SDS-PAGE was performed with 15% or 12% (w/v) gels for ethanol-soluble proteins or extracts containing AhA1 protein, respectively. Total protein amount loaded per well was 10 μg. After electrophoresis, gels were stained with Coomassie blue R-250 or used for Western blotting detection. Densitometric analysis of the target protein bands was performed using Image Studio Light software v.5.2 (LI-COR Biosciences, Lincoln, NE, USA).

Determination of Met and Cys

Sample hydrolysis. 100 mg of the tested sample was placed in an evaporation dish and 5 ml of an oxidizing mixture (hydrogen peroxide:formic acid=1:9) was added. The mixture was completely dried in a water bath at 60 °C, stirring occasionally. The dry residue was quantitatively transferred into a vial, and 10 ml of a 6 mol/dm^3 HCl solution was added. The sample hydrolysis was carried out in a thermostat at 110 °C for 18 h.

Amino acid modification reaction with phenylisothiocyanate (FITC). After cooling, the hydrolysate was filtered and 0.1 ml was taken to dry in the system of evaporation and concentration Smart Evaporator C1 (BioChromato, Fujisawa, Japan) at 60 °C. To the dried aliquot, 0.1 ml of a 0.1 mol/dm^3 Na_2CO_3 solution was added. Then, 0.35 ml of a solution of phenyl isothiocyanate in isopropanol (1:35) and 0.05 ml of distilled water were added. The mixture was incubated for 20 min at a room temperature and immediately dried at 60 °C. The dry residue was dissolved in 1 ml of distill water and centrifuged for 5 min at 10,000 rpm. The supernatant was used for injection into the chromatograph LC-20 Prominence (Shimadzu, Kyoto, Japan).

Measurement conditions. Elution mode - gradient. Component A - acetate buffer (0.06 mol/dm^3), pH 5.5. Component B – acetonitrile: isopropanol (99:1). Component C - acetate buffer (0.06 mol/dm^3), pH 4.05. The flow rate of the eluent is 1.2 ml/min. The detector wavelength is 254 nm. Column temperature 35 °C. The volume of the injected sample is 0.02 ml.

Time, min	Volume fraction of the component,%		
0.01	95	5	0
10.0	45	15	40
13.0	85	15	40
13.01	20	80	0
16.3	20	80	0
16.31	95	5	0
26.0	95	5	0

Determination of Asp, Glu, o-Pro, Ser, Gly, His, Arg, Thr, Ala, Pro, Tyr, Val, Ile, Leu, Phe, Trp, Lys

Sample hydrolysis. 100 mg of the tested sample was placed in a vial; 10 ml of a 6 mol/dm^3 HCl solution was added and hydrolyzed in a thermostat at 110 °C for 18 h.

Amino acid modification reaction with phenylisothiocyanate (FITC). Further, sample preparation was carried out as described above, with exception of NaOH (0.15 mol/dm^3) used as an alkaline component.

Measurement conditions. Elution mode - gradient. Component A - acetate buffer (0.06 mol/dm^3), pH 5.5. Component B - acetonitrile: isopropanol (99:1). Component C - acetate buffer (0.06 mol/dm^3), pH 4.05. The flow rate of the eluent is 1.2 ml/min. The detector wavelength is 254 nm. Column temperature 55 °C. The volume of the injected sample is 0.02 ml.

Time, min	Volume fraction of the component, %		
	Component A	Component B	Component C

0.01	96	4	0
10.0	37	11	52
13.0	88.5	11.5	0
21.0	80	20	0
22.0	58	22	20
24.0	0	24	76
32.0	0	33.5	66.5
32.01	20	80	0
35.3	20	80	0
35.31	97	3	0
41.3	97	3	0

Determination of Trp

100 mg of the tested sample was placed in a vial, 5 ml of a hot (80 °C) saturated solution of barium hydroxide was added and hydrolyzed in a thermostat at 110 °C for 18 h. Afterwards, the cooled hydrolysate was quantitatively transferred to a 50 ml volumetric flask and neutralized with a sulfuric acid solution (1.8 mol/dm^3). Then, the solution was brought to the mark with water and centrifuged. One ml of the supernatant was used for injection into the chromatograph.

Measurement conditions. Elution mode - gradient. Component A - acetate buffer (0.06 mol/dm^3), pH 5.5. Component B - acetonitrile: isopropanol (99:1). Component C - acetate buffer (0.06 mol/dm^3), pH 4.05. The flow rate of the eluent is 1.2 ml/min. The detector wavelength is 278 nm. Column temperature 50 °C. The volume of the injected sample is 0.02 ml.

Time, min	Volume fraction of the component, %		
	Component A	Component B	Component C
0.01	35	5	60
6.0	33	7	60
9.0	29	11	60
9.01	20	80	0
12.30	20	80	0
12.31	97	3	0
18.0	97	3	0
18.5	95	5	60
